# Immunological Characteristics and Properties of Glial Restricted Progenitors of Mice, Canine Primary Culture Suspensions, and Human QSV40 Immortalized Cell Lines for Prospective Therapies of Neurodegenerative Disorders

**DOI:** 10.1177/0963689719848355

**Published:** 2019-05-24

**Authors:** Aleksandra Klimczak, Urszula Kozłowska, Joanna Sanford, Piotr Walczak, Izabela Małysz-Cymborska, Maciej Kurpisz

**Affiliations:** 1 Institute of Human Genetics Polish Academy of Sciences, Poznan, Poland; 2 Hirszfeld Institute of Immunology and Experimental Therapy, Polish Academy of Sciences, Wroclaw, Poland; 3 VetRegen Laboratory and Bank of Stem Cells, Warsaw, Poland; 4 Department of Radiology and Radiological Science, Johns Hopkins School of Medicine, Baltimore, USA; 5 Department of Neurology and Neurosurgery, University of Warmia and Mazury, Olsztyn, Poland

**Keywords:** glial cells, glial progenitors, glial cell markers, cytokines

## Abstract

Neurodegeneration can be defined as a process in which neuronal structures and functions undergo changes leading to reduced neuronal survival and increased cell death in the central nervous system (CNS). Neuronal degeneration in specific regions of the CNS is a hallmark of many neurodegenerative disorders, and there is reliable proof that neural stem cells bring therapeutic benefits in treatment of neurological lesions. However, effective therapy with neural stem cells is associated with their biological properties. The assessment of immunological properties and comprehensive studies on the biology of glial restricted progenitors (GRP) are necessary prior to the application of these cells in humans. This study provides an in vitro characterization of the QSV40 glial human cell line, as well as murine and canine primary culture suspensions of GRPs and their mature, astrocytic forms using flow cytometry and immunohistochemical staining. Cytokines and chemokines released by GRPs were assessed by Multiplex ELISA. Some immunological differences observed among species suggest the necessity of reconsidering the pre-clinical model, and that careful testing of immunomodulatory strategies is required before cell transplantation into the CNS can be undertaken.

## Introduction

Biomedical research with subsequent clinical trials may lead to the development of new strategies to treat human neurodegenerative diseases that, at present, collect a grim harvest of death since the majority of them are untreatable^1^. Neurodegeneration is a process in which neuronal structure and functions are changed, leading to reduced neuronal survival and increased neuronal death in the central nervous system (CNS)^2^. Neuroinflammation initially constitutes a protective mechanism in the brain; its function is to signal repair of the damaged tissue, but overrepresentation of the inflammatory response has detrimental and inhibitory effects towards neuronal regeneration^2,3^. Currently, there are no therapeutic options that can induce neuronal regeneration in damaged or affected regions in the incredibly complex and sensitive bio-computer that is the CNS.

Neuronal degeneration in specific regions of the CNS is a hallmark of many neurodegenerative disorders, including amyotrophic lateral sclerosis (ALS), Parkinson’s disease or Alzheimer’s disease. In Alzheimer’s disease, neurodegeneration first involves the temporal lobes responsible for short-term memory, followed by, in a progressive stage of the disease, the parietal lobes responsible for long-term memory. In Parkinson’s disease, the degeneration process involves dopaminergic neurons in the substantia nigra^3^. ALS, also known as motor neuron disease, Lou Gherig’s disease, or the Charcot disease, is a neurological disorder that affects upper and lower motor neurons^4^. ALS has been classified as a genetic disorder (involving mutations in superoxide dismutase 1 (SOD1) and TAR DNA-binding protein 43 (TDP-43) genes as well as the TAR/FUS binding DNA factor and RNA proteins) only in 5–10% cases; however, the majority (90–95%) of subjects do not carry these genomic mutations and thus environmental factors responsible for disease development must also be considered^1,4–6^. Different strategies involving cellular therapies using different types of stem or progenitor cells have been tested to treat neurodegenerative diseases^7^. To date, there are no specific effective pharmacologic treatments to repair damaged neurons and induce neuroregeneration processes. Transplantation into the CNS of cells of physiological origin, such as neural precursor cells, may help replace damaged sites in either a direct and/or indirect fashion. However, this potentially promising strategy is still experimental due to poor graft viability and survival. The exact reasons for cell graft death in CNS are not fully understood. A better understanding of the unique character of the CNS and its interaction with the immunological system are crucial in achieving this goal.

Glial restricted progenitors (GRPs) are multipotent cells of ectodermal origin. They are restricted to resume three possible functions after maturation: they may differentiate and form astrocytes type I, astrocytes type II, or oligodendrocytes^8–10^. Astrocytes participate in the control of correct ionic balance in the neuronal vicinity. They also form the blood–brain barrier (BBB), and influence immune response by direct cooperation with microglia. Astrocytes maintain neurotransmitter metabolism and nutrition of neurons, as they have a natural ability to store glycogen, and also participate in the process of myelination^11–14^. Transplanted GRP cells are able to migrate and assume a position in close proximity to injured motor neurons, differentiate into astrocytes (60%) and oligodendrocytes (10%) and may repair damaged neurons, which results in improvement of locomotion functions of the subject^15^.

Here, we present the results of immunological characterization of GRPs, which, to date, seem to be the best target for a therapeutic option in neurodegenerative diseases. Physiological differences were observed among GRPs obtained from different species: mouse, dog, and human. Comprehensive characterisation of GRPs will be necessary to create appropriate strategies for graft survival and the development of satisfactory and safe cellular therapies for CNS treatment.

## Materials and Methods

### GRP Cells

Murine GRP cells were obtained as a gift from the Department of Radiology and Radiological Science, Johns Hopkins School of Medicine (Baltimore, MD, USA), while canine GRP cells suspensions were provided by Department of Neurology and Neurosurgery, University of Warmia and Mazury, Olsztyn, Poland. Animal tissue experimentation was carried out according to EU Directive 2010/63/EU, and was accepted by the Local Ethical Committee for Animal Experimentation, Poznan University of Life Sciences (Resolution No 12/2017).

Murine GRPs cells were isolated as previously described^16^. Spinal cords were dissected from mice^Luc+/PLP/GFP+^ between E12.5 and E14 and plated on a Petri dish in DMEM/F12 medium (Gibco, Gaithersburg, MD, USA). Tissue samples were then incubated in pre-warmed TrypLE Express (Gibco) with 10mg/mL DNase-1 (A&A Biotechnology, Gdansk, Poland) for 10–12 min, gently agitated, and incubated for a further 10 min. Next, 5 mL of GRP medium was added and spun down at 1000 rpm for 5 min. The pellet of cells obtained was resuspended in 10 mL of GRP medium with 10mg/mL of DNase, and incubated at 37°C in humidified incubator with 5% CO_2_ for 10 min. The pellet was then mechanically agitated again and spun down at 1000 rpm for 5 min, resuspended in 10 mL of GRP medium, plated on coated PLL/Laminin 25 mL flasks in a humidified incubator at 37°C with 5% CO_2_, and grown until reaching 80% confluence.

Canine GRPs cells were isolated from fetuses originating from abortive sterilization. The owners of the dogs subjected to sterilization provided written consent to transfer material for cell isolation. Isolation of canine GRPs was based on the above-described procedure for murine GRP isolation. Brains and spinal cords were dissected from canine fetuses between E32 and E37. Briefly, the tissue was incubated in pre-warmed TrypLE Express (Gibco) with 10 mg/mL DNase-1 (A&A Biotechnology) for 10–12 min, gently triturated, and incubated at 37°C for 10 min. Next, 5 mL of GRP medium was added, and the suspension was centrifuged at 1000 rpm for 5 min. The pellet was resuspended in 10 mL of GRP medium with 10 mg/mL of DNase, and incubated at 37°C in a humidified atmosphere with 5% CO_2_ for 10 min. The pellet was then triturated again and centrifuged at 1000 rpm for 5 min, resuspended in 10 mL of GRP medium, and plated on coated PLL/Laminin 25-mL flasks in 37°C in a humidified incubator with 5% CO_2_. Cells were cultured for 5–10 days (1–2 passages) in GRP medium with basic fibroblast growth factor (bFGF), harvested with TrypLE Express (Gibco), cryopreserved in ATCC medium (LGC Standards, Teddington, UK), and stored in vapor phase liquid nitrogen until analysis.

### GRP Culture

Human GRP cells were obtained as a gift from the Department of Radiology and Radiological Science, John Hopkins University, as a QSV40 transformed cell line. Culture flasks were coated with poly-l-lysine and laminin. Cells were cultured while maintained in DMEM F12 medium (Gibco) supplemented with 1% bovine serum albumin (BSA; Abcam, Cambridge, UK), and bFGF (Peprotech, Rocky Hill, NJ, USA) supplements at 5% CO_2_ atmosphere at 37°C. Cell passages were performed according to culture fluency – mostly between 6 and 10 days of in vitro culture at a density of 25,000 cells per 1 cm^2^.

Flow cytometry and immunofluorescence were applied to evaluate neurogenic (A2B5, NG2, GFAP, Nestin, PSA-NCAM), immunogenetic (MHC class I, MHC class II), and costimulatory (CD28, CD40, CD80, CD154) molecules of the obtained GRP cells of murine, canine, and human origin (see Table 1 for detailed marker characteristics, abbreviations, and antigenic description).

**Table 1.** Antibody Characteristics.

Neural Markers	IMMUNOFLUORESCENT STAINING					
	Marker	Isotype	Company	Reactivity	Dilution	
	Nestin	IgG1	Abcam	M	1:20	
	Nestin	IgG	Novus	Hu, Dg	1:200	
	GFAP	IgG	Abcam	Ma	1:1000	
	Nestin	IgG1 κ	R&D Systems	Hu	1:200	
	PSA-NCAM	IgM	Merck	Ma	1:200	
	A2B5	IgM	Merck	Ma	1:30	
	NG2	IgG1	Abcam	M	1:100	
	FLOW CYTOMETRY					
	Marker	Isotype	Company	Reactivity	Fluorochrome	Dilution
	GFAP	IgG2b κ	BD Pharmingen	Ma	AF 647nm	1:25
	Nestin	IgG1 κ	R&D Systems	Hu	---	1:25
	NG2	IgG1	Abcam	M, Hu	---	1:20
	Mouse IgG1 κ, Isotype control	IgG1 κ control	Abcam	---	---	1:50
	Mouse IgG2b κ, Isotype control	IgG2b κ control	BD Pharmingen	---	AF 647nm	1:10
	A2B5	IgM	Miltenyi Biotec	M, Hu	APC	1:20
	PSA-NCAM	IgM	Miltenyi Biotec	Ma	APC	1:20
	Mouse IgM, Isotype control	IgM control	Miltenyi Biotec	---	APC	1:20
Immunological Markers	IMMUNOFLUORESCENT STAINING					
	Marker	Isotype	Company	Reactivity	Dilution	
	MHC-II	IgG2b	Abcam	M	1:50	
	MHC-II	IgG2a	Abcam	Hu	1:200	
	MHC-I	IgG	Abcam	M, Hu	1:50	
	MHC-I	IgG	Abcam	Hu	1:200	
	FLOW CYTOMETRY					
	Marker	Isotype	Company	Reactivity	Fluorochrome	Dilution
	MHC-II	IgG2a	Bio-Rad	Dg	FITC	1:10
	Rat IgG2a, Isotype control	IgG2a control	Bio-Rad	---	FITC	
	MHC-II	IgG2a	Bio-Rad	Hu	FITC	1:10
	Rat IgG2a, Isotype control	IgG2a control	Bio-Rad	---	FITC	1:10
	MHC-II	IgG2b κ	Abcam	M	APC	1:25
	Rat IgG2b κ, Isotype control	IgG2b κ control	Abcam	---	APC	1:25
	MHC-I	IgG2a	Bio-Rad	Hu	AF 647nm	1:10
	Mouse IgG2a, Isotype control	IgG2a control	Bio-Rad	---	AF 647nm	1:10
	CD28	IgG2 λ	BD Pharmingen	M	PE	1:10
	Hamster IgG2 λ, Isotype control	IgG2 λ control	BD Pharmingen	---	PE	1:10
	CD154	IgG3 κ	BD Pharmingen	M	PE	1:10
	PE Hamster IgG3, κ	IgG3 κ control	BD Pharmingen	---	PE	1:10
	CD40	IgM κ	BD Pharmingen	M	AF 647nm	1:10
	AF 647 nm Hamster IgM, λ1 Isotype control	IgM λ control	BD Pharmingen	---	AF 647nm	1:10
	CD80	IgG2 κ	BD Pharmingen	M. Dg	APC	1:10
	Marker	Isotype	Company	Reactivity	Fluorochrome	Dilution
Immunological Markers	APC Hamster IgG2, κ Isotype control	IgG2 κ control	BD Pharmingen	---	APC	1:10
	CD28	IgG1 κ	BD Pharmingen	Hu	FITC	1:10
	CD40	IgG1 κ	BD Pharmingen	Hu	FITC	1:10
	CD154	IgG1 κ	BD Pharmingen	Hu	FITC	1:10
	CD80	IgG1 κ	BD Pharmingen	Hu	FITC	1:10
	FITC Mouse IgG1, κ Isotype control	IgG1 κ control	BD Pharmingen	Hu	FITC	1:10
	CD28	IgG1 K	Thermo Fisher	Dg	APC	1:10
	Mouse IgG1 K Isotype Control eFluor® 660	IgG1 K control	Thermo Fisher	---	eFluor® 660	1:10
	CD40	Surface	Bio-Rad	Hu, Dg	AF 647nm	1:10
	Mouse IgG2a, Isotype control	IgG2a control	Bio-Rad	---	AF 647nm	1:10

Hu: human; Dg: dog; M: murine; Ma: Mammals; AF: Alexa Fluor; Nestin: neural stem cell marker; PSA-NCAM: Polysialylated–Neural Cell Adhesion Molecule (marker of neural precursor cells); A2B5: marker of neural progenitor cells; GRP cells: type II astrocytes; GFAP: Glial Fibrillary Acidic Protein (astrocyte marker, glial cell marker); NG2: Nerve/Glial antigen 2 (marker of oligodendrocyte precursor cell (OPCs)); CD28, CD 40, CD 80, CD154: costimulatory molecules; MHC- I: main histocompatibility complex Class I antigens; MHC-II: main histocompatibility complex Class II antigens.

### Preparation of Blood Cells for Control

Murine blood (1 mL) was collected from C57BL/6 mice (*n* = 4) by cardiac puncture, placed in a tube with 50 µl of 2% EDTA solution, and agitated. Human blood samples were collected from four healthy volunteers, and dog blood samples (*n* = 4) were collected into EDTA tubes. Samples were processed immediately after collection. Blood samples were diluted 1:1 with PBS; 2 mL of the diluted samples were then placed gently into 15 mL Falcon tubes filled with 4 mL of Histopaque. Samples were centrifuged for 20 min at 400 *g* at room temperature (RT). After centrifugation, the upper layer was gently aspirated, and the middle mononuclear cell layer was collected. The cells were washed in 5 mL of PBS, and centrifuged for 5 min at 300 *g*. To remove red blood cells from collected samples, the pellet was incubated 5 min at RT in 1 mL of 0.1% saponin. After incubation, 10 mL of PBS was added, and the suspension was centrifuged for 10 min at 300 *g* speed. When treatment with saponin was insufficient and red blood cells were still visible in the pellet, the incubation with saponin was repeated and the cells washed with PBS. PBS was carefully removed and pellet resuspended to obtain a final concentration of 2 × 10^5^ cells/50 µL.

### Flow Cytometry Analysis

**Fig 1.**
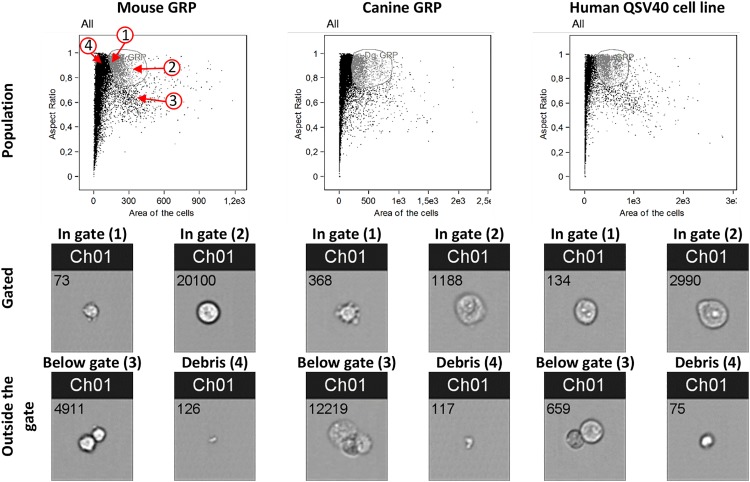
Aspect ratio and area of cell scatter of mouse and canine GRPs and the human QSV40 cell line. The grey circle represents cell populations gated for the analysis. Spots 1 and 2 on the scatter present representative photos of analyzed cells within gates. Spot 3 presents cells not included in the gates (duplex, triplex or multiplex cell clusters). Spot 4 presents an area outside the gate, where cell debris is present. The same code was applied to GRPs cells from all species. Ch01 is a bright field channel from which cell photos were acquired. The number on each photograph is an ordinal number of acquired cell in analysis file. Analysis was performed on Amnis cytometer using IDEAS Application software v 6.0.

Cells were detached from the surface of the cell culture flask using TrypLE Express digestion at 37°C for 3 min. Cells were centrifuged for 5 min at 1000 rpm. Staining for surface markers (CD28, CD40, CD80, CD154, PSA-NCAM, A2B5, and MHC class I and class II) was carried out on fresh, living cells. Cell viability was assessed by Trypan Blue exclusion. Primary antibody was added to 50 µL of cell suspension in PBS (4 x 10^5^ cells), and incubated 30 min on ice protected from light. For staining of intracellular markers (GFAP, Nestin), cells were fixed for 20 min at 4°C in 250 µL of BD Cytoperm/Cytofix (BD Biosciences, San Jose, CA, USA). After incubation, cells were centrifuged at 200 *g* for 5 min, resuspended in 500 µL of Perm Wash Buffer (BD Biosciences), and again centrifuged at 200 *g* for 5 min. The pellet was resuspended in 50 µL of PBS, primary antibody was added and incubated for 30 min on ice in the dark. After incubation with primary antibody, 1.3 mL of PBS was added to block the reaction. All the antibodies and isotype controls were conjugated with fluorochrome (see Table 1). For each antibody, depending on its isotype and the fluorochrome conjugated, the proper isotype control staining was prepared for all types of analyzed cells. The dilutions of each primary antibody and isotype controls used are presented in Table 1. After incubation, the cells were washed twice in PBS and centrifuged 5 min at 200 *g*, resuspended in 50 µL of PBS, and immediately analyzed with an Amnis cytometer. Data were analyzed using IDEAS Application software v 6.0. GRPs from the studied species had different sizes and gating strategies, as illustrated in Fig 1.

### Multiplex ELISA

Supernatants collected after each passage when the cells reached 80% confluence were centrifuged for 10 min at 200 *g*. Supernatants were aspirated, aliquoted and stored at –80°C until further analysis. On the day of analysis, samples were thawed on ice and vortexed carefully. For murine cells and human cell line, analysis points were made at passages P1–P10. For the human QSV40 cell line, supernatant samples were assayed for 27 cytokines (Bioplex panel; Bio-Rad, Hercules, CA, USA) and trophic factors: FGF basic, Eotaxin, G-CSF, GM-CSF, IFN-γ, IL-1β, IL-1ra, IL-2, IL-4, IL-5, IL-6, IL-7, IL-8, IL-9, IL-10, IL-12 (p70), IL-13, IL-15, IL-17, IP-10, MCP-1 (MCAF), MIP-1α, MIP-1β, PDGF-BB, RANTES, TNF-α, and VEGF. For mice GRP, supernatants were sampled for 23 cytokines (Bioplex panel; Bio-Rad) for the following analyses: IL-1α, IL-1β, IL-2, IL-3, IL-4, IL-5, IL-6, IL-9, IL-10, IL-12(p40), IL-12(p70), IL-13, IL-17, Eotaxin, G-CSF, GM-CSF, IFN-γ, KC, MCP-1, MIP-1α, MIP-1β, RANTES, and TNF-α. For canine GRP, supernatants were tested for 13 cytokines (Milliplex panel; Merck, Burlington, MA, USA), including GM-CSF, IFN-y, IL-2, IL-6, IL-7, IL-8, IL-15, IP-10, KC-like, IL-10, MCP-1, and TNFa. These analyses were performed according to the manufacturers’ protocols. All canine supernatant samples were analyzed in triplicate, while human cell line QSV40 and murine GRP cells supernatants were tested in duplicate. Culture media were used as a diluent for protein standards attached to kits. Supernatants from human and murine GRP culture were recorded with a BioPlex 200 instrument, and analyzed with Bio Plex Manager Software. The Milliplex panel with canine GRP supernatants was read using a Luminex 200 system. The data are presented in selected passages that respond to early, mid-, and late-passage for human and murine cells P2, P6, and P10, and for canine GRP P0, P2, and P4, respectively (canine GRPs decrease proliferative activity after P5).

#### Immunofluorescence staining

Cells were placed in 24-well plates coated with poly-l-lysine and laminin. After assuming their native shape, cells were fixed in 4% PFA for 40 min at RT, then incubated in a mixture of 1% BSA (Abcam), 10% goat serum (Abcam), and 0.1% Tween-20 to decrease the risk of unspecific binding and permeabilize the cell membranes if necessary. Time of incubation and dilution depended on antibody characteristics (Table 1). After incubation with primary antibody, secondary antibody was applied for 1 h (Alexa Fluor, 1:600) at RT [red fluorochrome Alexa Fluor 594 nm, green fluorochrome Alexa Fluor 488 nm]. Nuclei were stained with DAPI. Immunostainings were analyzed using a fluorescent microscope (Leica, Wetzlar, Germany) equipped with a monocamera and LAS X software for picture acquisition.

#### Differentiation assay

GRPs were differentiated to astrocytes after 3–5 days incubation in GRP medium enriched with 15% fetal bovine serum (FBS; BioWest, Nuaille, France). The cells were examined for the presence of GFAP by flow cytometry and immunocytochemistry as described earlier.

## Results

### Murine GRPs

#### Phenotype

**Fig 2.**
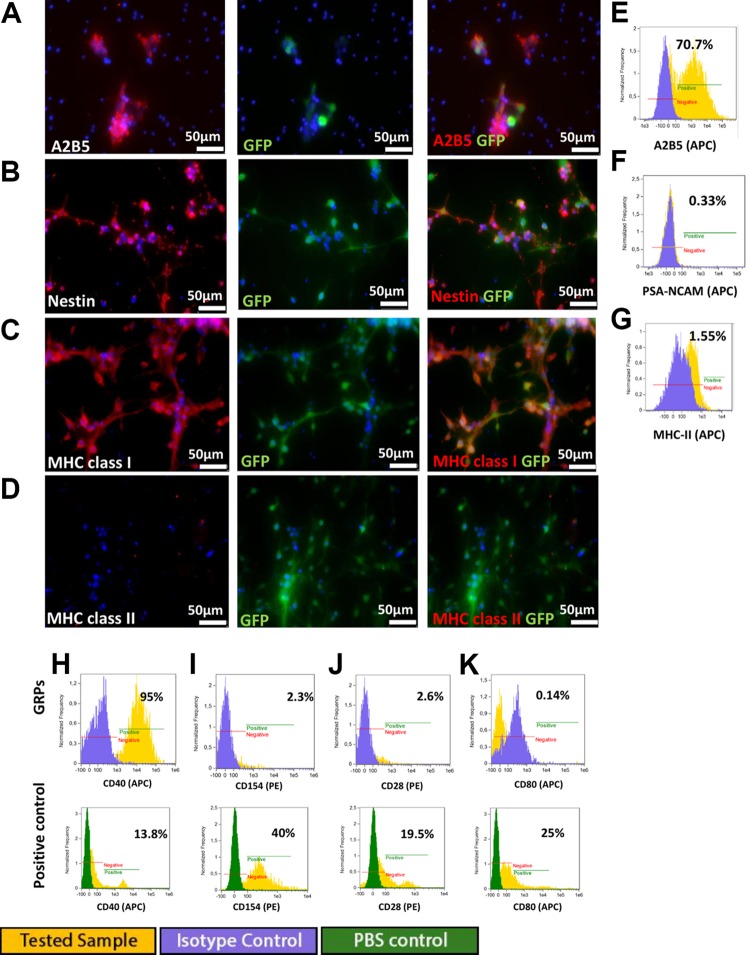
Immunofluorescence staining of murine GRP cells: (A) expression of A2B5 in half of the cell population, (B) strong expression of nestin in the majority of cells studied, (C) strong expression of MHC class I in the majority of cells studied, (D) no expression of MHC class II. Flow cytometry analysis showed: (E) A2B5 expression at 70% of analyzed cells and low expression of (F) neural cell marker PSA-NCAM (0.33%) and (G) MHC class II (1.55%). Analysis of costimulatory molecules expression, with their positive control (blood cells): (H) expression of CD40 at 95%, (I) CD154 at 23%, (J) CD28 at 2.6%, and (K) CD80 at 0.14%.

Murine GRPs express A2B5 and nestin markers specific for neural progenitors and are negative for GFAP and PSA-NCAM (Fig 2 A, B, E, F). Analysis of the immunogenic properties of murine GRPs showed that these cells are marked by strong MHC class I expression and lack of expression of MHC class II antigens (Fig 1 C, D, G). Costimulatory molecules were represented by CD40-positive staining on a significant proportion of cells (range 6.3–96%), whereas expression of the other co-stimulatory molecules examined (CD28, CD80, and CD154) was observed at a very low level (range 0.1–2.3%) (Fig 2 H, I, J, K).

Immunofluorescent staining and flow cytometry revealed that murine GRPs cells preserve a stable pro-neurogenic phenotype in long-term in vitro culture up to P10. This conclusion followed from the observation of nestin and A2B5 expression and a lack of astrocyte marker – GFAP or neural cell marker PSA-NCAM.

#### Astrocyte differentiation

**Fig 3.**
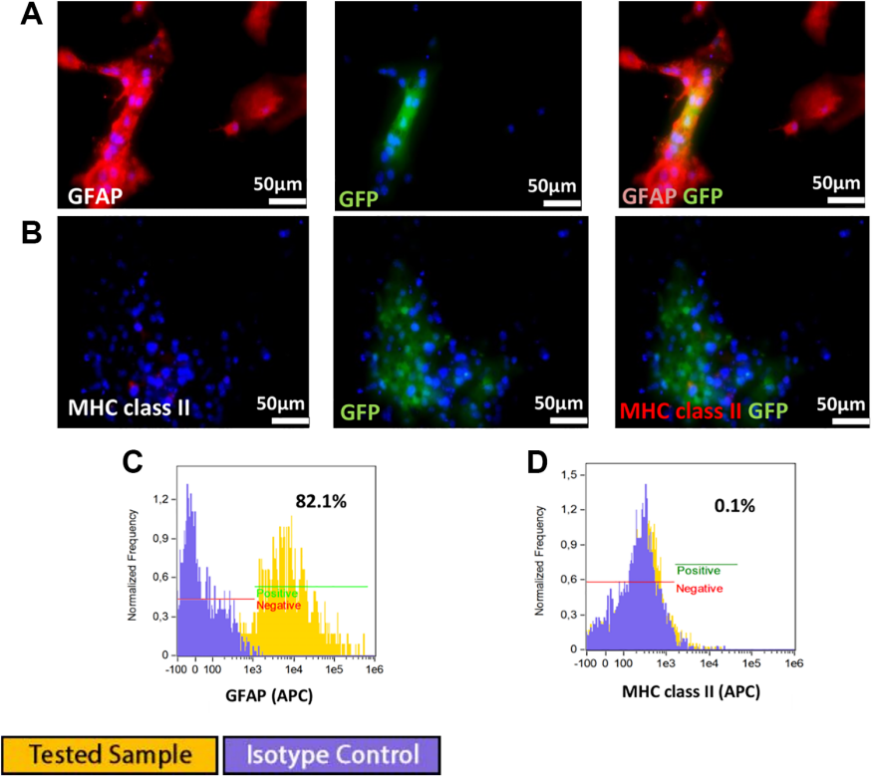
(A) GRP differentiation into astrocytes was confirmed by positive GFAP staining. 9B) Astrocyte differentiation had no influence on MHC class II expression. (C) flow cytometry analysis confirmed that 82% of cells were GFAP positive. (D) lack of MHC class II expression.

Murine GRP cells efficiently differentiated into astrocytes in the presence of 15% FBS in culture medium. At 5 days of in vitro culture, differentiation of GRPs cells into astrocytes was confirmed by the presence of GFAP expression (Fig 3 A, C) using immunofluorescence and flow cytometry. Flow cytometry analysis revealed 82% GFAP-positive cells (Fig 3C). Astrocyte differentiation had no influence on MHC class II expression under in vitro culture conditions (Fig 3 B, D).

### Canine GRPs

#### Phenotype

**Fig 4.**
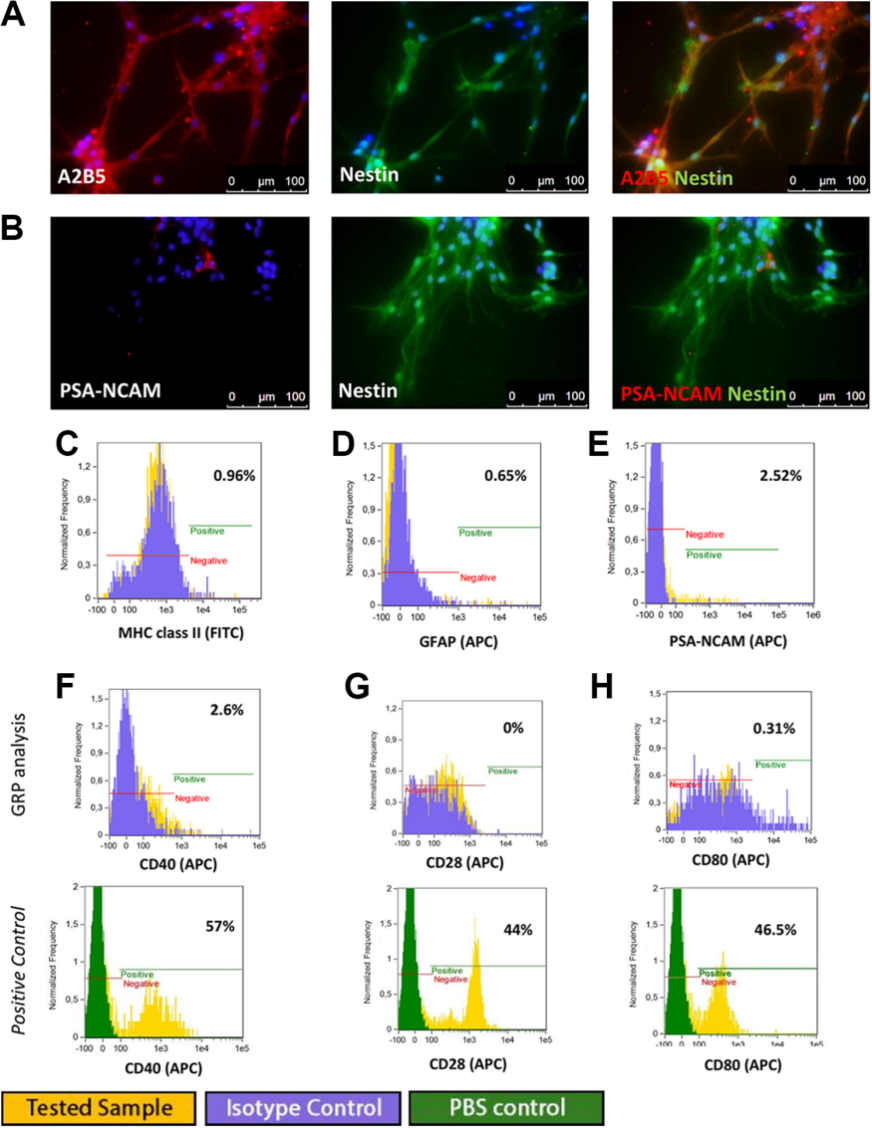
Immunofluorescence staining of canine glial restricted progenitors: (A) Co-expression of nestin and A2B5, (B) rare PSA-NCAM positive cells situated beneath nestin-positive GRPs. Flow cytometry analysis: (C) Weak expression of class II MHC, (D) no expression of GFAP, and (E) low expression of PSA-NCAM (2.52%). Flow cytometry analysis for costimulatory molecules expression, with positive control (blood cells) revealed: (F) Expression of CD40 at 2.6%, (G) no expression of CD28, (H) weak expression of CD80 at 0.31%.

Canine GRP cells revealed expression of nestin and A2B5 (Fig 4A). Single PSA-NCAM positive cells were observed using immunocytochemistry (Fig 4B). Canine GRP cells exhibited weak expression of MHC class II (0.96%) (Fig 4C). A lack of GFAP-positive cells was confirmed by flow cytometry (Fig 4D). Flow cytometry confirmed a PSA-NCAM-positive signal in 2.52% of cells in passage 1 (Fig 4E). GRPs were also characterized by the presence of costimulatory molecules CD28, CD40, and CD80, and a low number of CD40-positive cells of canine GRP (2.6%) was detected (Fig 4F).

#### Astrocyte differentiation of canine GRPs

**Fig 5.**
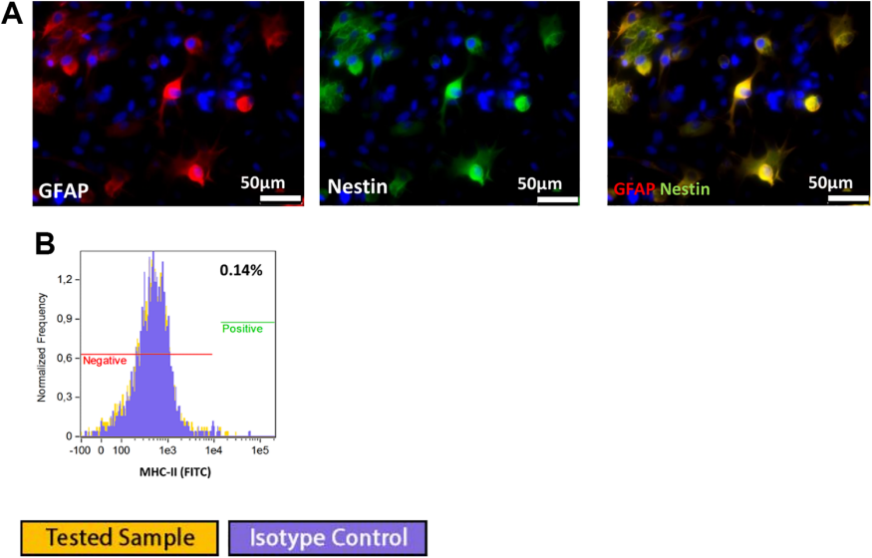
Canine cell differentiation into astrocytes was confirmed by (A) positive GFAP and nestin staining. (B) GFP differentiation into astrocytes had no influence on MHC class II expression.

Canine GRPs cultured in media enriched with 15% FBS, dedicated for astrocyte differentiation, successfully differentiated into astrocytes as confirmed by expression of GFAP and nestin (Fig 5A). Cytometric analysis revealed a lack of MHC class II expression on differentiated canine GRPs (Fig 5B).

#### Human QSV40 GRP cell line

**Fig 6.**
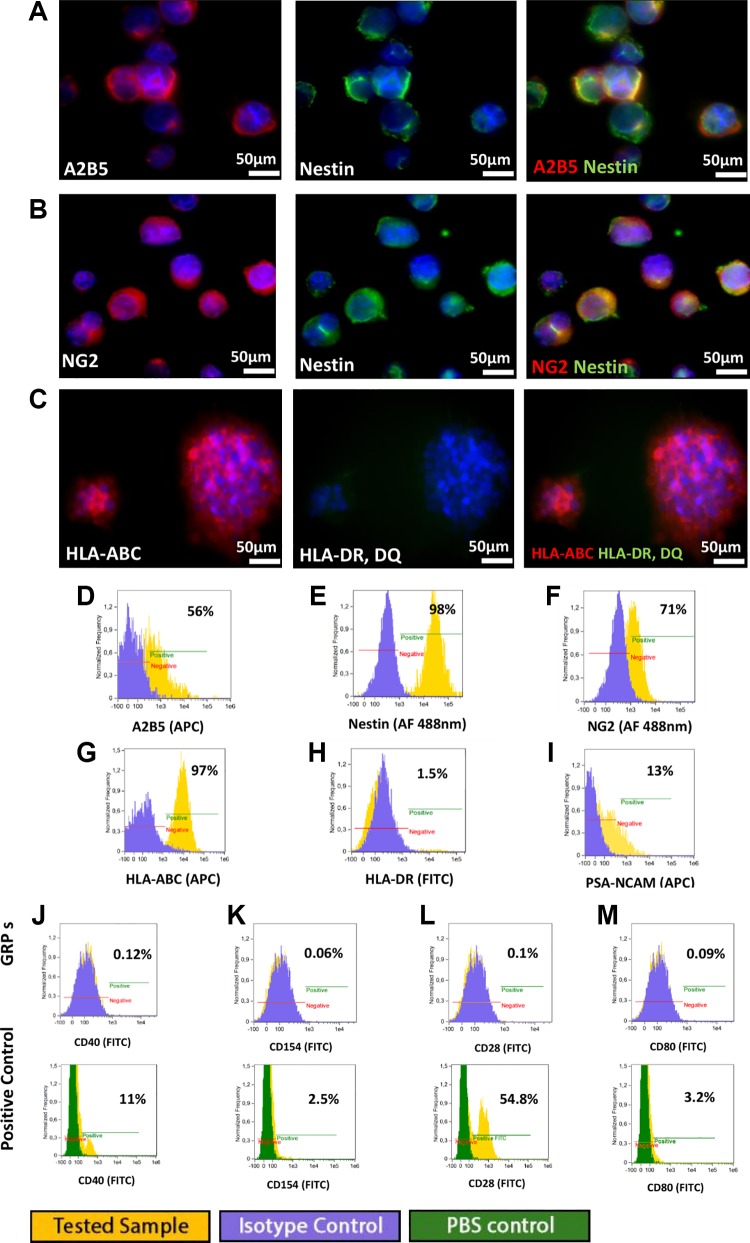
Immunofluorescence staining and flow cytometry analysis of human QSV40 glial cell line. QSV40 cells co-expressed markers: (A) A2B5 and nestin, (B) NG2 and nestin specific for glial-restricted progenitors. (C) QSV40 cell line expressed HLA-ABC but no expression of HLA class II was observed. Cytometric analysis revealed expression of (D) A2B5 in 56% of cell population, (E) high expression of nestin (98%), (F) expression of NG2 in 71% of cell population, (G) expression of HLA-ABC in 97% of cells, (H) low expression of HLA-DR 1.5%, (I) and weak expression of PSA NCAM (13%). Cytometric analysis for costimulatory molecules expression, with positive control revealed: (J) CD40 at 0.12%, (K) CD154 at 0.06%, (L) CD28 at 0.1%, and (M) CD80 0.09%

The human QSV40 GRPs cell line was characterized by progenitor markers characteristics of the glial restricted phenotype. Cells were positive for expression of A2B5 nestin and NG2 (Fig 6 A, B, D, E, F). The QSV40 cell line is phenotypically stable, and changes in phenotype were not observed after subsequent passages up to P10. A 13% population of cells expressed PSA-NCAM (Fig 6 I). Analysis of HLA class I and class II antigens using immunofluorescence and flow cytometry revealed strong expression of HLA-ABC class I antigen and a lack of HLA-DR antigens (Fig 6 C, G, H). Co-stimulatory molecules characterized by CD40, CD154, CD28, CD80 were absent in the QSV40 cell line (Fig 6 J, K, L, M).

#### Astrocyte differentiation

**Fig 7.**
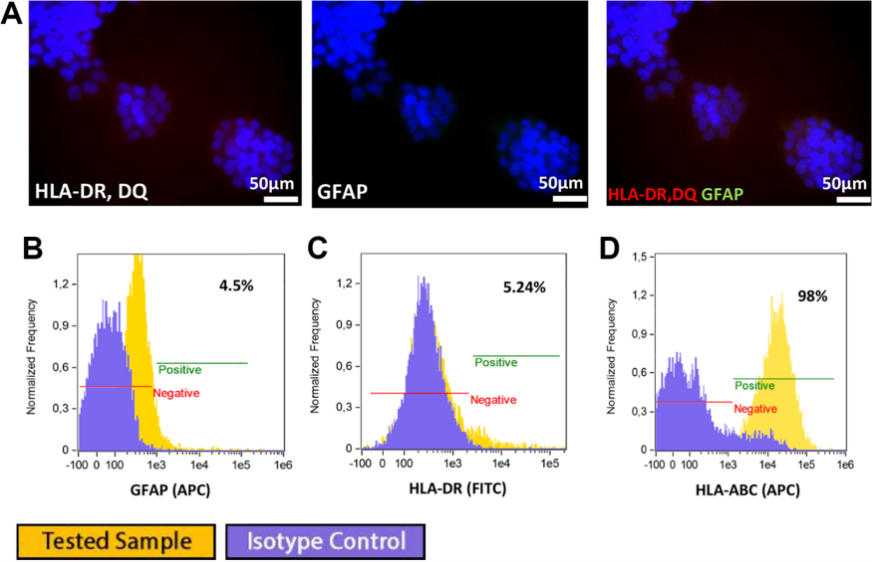
Immunofluorescence staining revealed lack of astrocytic differentiation of immortalized human cell line. (A) Low expression of GFAP or HLA-DR, DQ was observed. Flow cytometry analysis showed: (B) weak expression of GFAP in 4.5% cell population, (C) weak expression of HLA-DR (5.2%), (D) no changes were observed in HLA-ABC expression (98%).

QSV40 cell cultures in medium enriched with 15% FBS, dedicated for astrocyte differentiation, were unsatisfactory in the case of the human immortalized QSV40 cell line. Immunofluorescence staining and flow cytometry analysis revealed low expression of GFAP (4.5% in Fig 7 A, B). Cultures in astrocyte differentiation medium had no influence on HLA-ABC class I expression in the QSV40 cell line (Fig 7 D). Low expression of HLA-DR antigens (5.24%) was detected (Fig 7 C).

#### Cytokines and trophic factors secreted by GRPs

**Fig 8.**
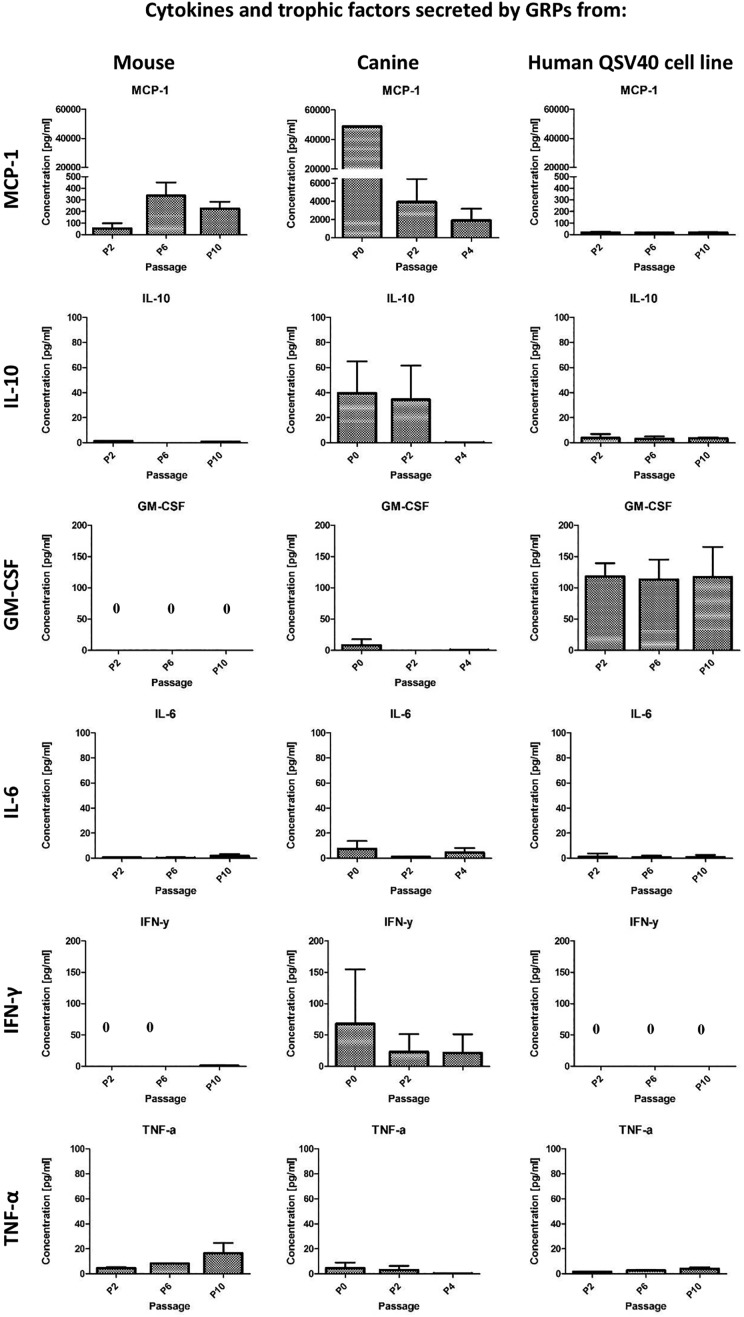
Multiplex ELISA for cytokines and chemokines secretion by mouse and canine GRPs and human QSV40 cell line measured in supernatants after in vitro cell culture. Analysis was performed at passage P2, P6, and P10 for mouse GRPs and human QSV40 cell line, and at P0, P2, and P4 for canine GRPs. Murine GRPs as well as canine GRPs, but not human QSV40 GRP cell line, were able to secrete high levels of MCP-1. The highest concentration of IL-10 was measured in supernatant after canine GRP in vitro culture. Human QSV40 cell line was able to secrete the highest levels of GM-CSF. GRP from all species were able to secrete very low levels of IL-6. IFNγ was detected in supernatants after canine GRP in vitro culture. GRP from all species were able to secrete low levels of TNF-α.

GRP cells were able to secrete some immunomodulatory factors. Expression of cytokines and chemokines varied between species and also between early and late passages. The levels of selected cytokines and trophic factors are presented in Fig 8. Murine and canine cells were able to secrete high levels of Monocyte Chemoattractant Protein-1 (MCP-1). In murine GRPs supernatants, MCP-1 levels varied between passages; the lowest average concentration (54.80 pg/mL) was observed at passage P2, whereas the highest concentration (336.25 pg/mL) was detected at passage P6. However, the highest concentrations of MCP-1 were observed in supernatants after in vitro culture of canine GRP, with levels at passage P1 assessed at 48,728.38 pg/mL, decreasing during subsequent passages to, at late passage P10, levels of 1879.58 pg/mL. In contrast, the human QSV40 cell line was characterized by the lowest MCP-1 secretion of all of the species examined. The lowest observed concentration (16.43 pg/mL) was at passage P6 and the highest at passage P10 (18.86 pg/mL).

The levels of IL-10 in supernatants were very low for murine GRP and the human QSV40 cell line. The highest concentration was observed at passage P2 (1.29 pg/mL) for murine cells and also at passage P2 for human QSV40 cell line (3.8 pg/mL). In contrast, in canine GRP cells, IL-10 demonstrated the highest concentration, reaching 39.51 pg/mL at passage P1, and decreasing to below the standard curve at passage P4 (0.2 pg/mL).

Differences between GM-CSF secretions were also substantial between species. Only the human cell line secreted high amounts of GM-CSF, which was rather stable between passages (113.21 pg/mL–117.98 pg/mL) during the period of observation. Very low levels of GM-CSF were detectable after in vitro culture of canine GRP; the concentration was assessed at 8.09 pg/mL in P1, decreasing to 0.1 pg/mL and 0.23 pg/mL in passages P2 and P4, respectively. GM-CSF after culture of murine GRP cells was undetectable; however, G-CSF was seen at low levels (2.35 pg/mL in P2, 0.67 pg/mL in P10) (data not shown).

IL-6 was detectable in supernatants from all species examined; however, the level was low. The highest observed concentration was after in vitro culture of canine GRP at passage P0 (8.27 pg/mL), decreasing to 1.03 pg/mL at passage P2 (which was below the detection point). IL-6 levels in supernatants from mouse GRP varied between 0.47 pg/mL at P1 and 1.8 pg/mL at passage P10. After in vitro culture human QSV40 cells, IL-6 concentration was rather stable at low level (0.94 pg/mL–0.68 pg/mL), with the highest measured level at P2.

Low concentrations of IFN-γ were observed after murine cell culture only at passage P10 (1.08 pg/mL), with higher concentrations being observed after canine GRP culture (67.74 pg/mL at P0 and 21.7 pg/mL at P4). IFN-γ was undetectable in supernatants after human QSV40 cell line culture.

GRP from all species were able to secrete low levels of TNF-α. The range for murine GRP was 4.61 pg/mL at P2 and 16.37 pg/mL at P10. For canine GRP, TNF-α was assessed at 3.87 pg/mL at passage P0 and 0.36 pg/mL at passage P4. For the human QSV40 cell line, TNF-α was detected for 1.61 pg/mL at P2 and 4.23 pg/mL at P10 (Fig 8).

Other analyzed cytokines and trophic factors are not illustrated in Fig 8 because commercial kits used for Multiplex ELISA for different species differ in the content examined. Canine GRP and the human QSV40 line were able to secrete IL-8. Unfortunately, the murine screening kit did not include IL-8 analyte. In canine GRP, supernatants IL-8 levels were high, ranging from 1036.71 pg/mL at P2 to 1961.79 pg/mL at passage P4. Elevated levels of IL-8 were also observed in supernatants from the human cell line QSV40. The lowest concentration of IL-8 was detected at the middle passage P6 (93.13 pg/mL) and the highest (273.31 pg/mL) at passage P10. Low concentrations of IL-4 were observed after in vitro culture of murine (1.25–1.36 pg/mL) and human QSV40 (0.16–0.30 pg/mL) cell lines.

Other examined cytokines and trophic factors included in commercial kits were at very low levels or undetectable. Levels of IL-12 were out of range in murine supernatant, but low levels were present after in vitro culture of the human cell line QSV40 (10.22–23.29 pg/mL). Low concentrations of IL-13 were present in murine and human supernatants (4.02– 18.48 pg/mL and 0.51–1.24 pg/mL, respectively). IL-15 was present in supernatants after in vitro culture of canine (0.55–7.01 pg/mL) and human (2.12–2.23 pg/mL) GRP. IL-17 was detectable in supernatants after in vitro culture of murine (1.54–2.20 pg/mL) and human (10.9–17.16 pg/mL) GRPs. IP-10 was present in supernatants after in vitro culture of the human cell line (2.21–4.29 pg/mL) and in higher concentrations also after canine GRP cell culture (37.90–124.39 pg/mL). Low concentrations of MIP-1α were detected after murine (0.26–3.0 pg/mL) as well as after human cell line QSV40 in vitro culture only at passage P10 (0.23 pg/mL). MIP-1β was beyond the range of detection in murine supernatants; however, this chemokine was detectable at low concentration in single samples (0.79–2.3 pg/mL). Low levels of MIP-1β were also detected after in vitro culture of human QSV40 cells at P10 (0.82 pg/mL). RANTES was detectable only at passage P2 after murine GRP cell culture (0.87 pg/mL); however, very low levels were also present after human cell line in vitro culture (0.08–0.16 pg/mL). The human QSV40 cell line was also able to secrete low levels of PDGF-bb (3.71–4.55 pg/mL).

## Discussion

The regenerative potential of the CNS is extremely limited and neurodegenerative diseases still pose unsolved clinical and social problems. Cellular therapies applied to the sites of CNS injury could potentially recover function at the damaged site. Stem-cell-based therapies have been developed extensively in preclinical studies and clinical trials for ALS; however, the best source of stem cells for effective therapy and route of delivery is still debated^7,17^. Experimental studies with stem cell therapies have been developed widely in small animal models. Previous studies on glial restricted progenitors for therapy of neurodegenerative disorders yielded promising results^18–21^. In 2008, Leapore’s team observed a reduction in microgliosis after successful astrocytic differentiation of GRPs transplanted into the respiratory motor neuron tract in SOD1^G93A^ in a rat model. This procedure resulted in a marked delay in limbs and respiratory tract damage^21^. Other studies on the therapeutic potential of huGRPs transplanted into spinal cord dorsal white matter in a rat model with focal inflammatory demyelination were introduced in 2011 by Walczak’s group. The latter authors observed migration of huGRP injected into the spinal cord; however, despite the presence of low numbers of cells with an oligodendrocyte precursor cell phenotype, the rate of remyelinization in adult rat spinal cord was low. In contrast, in another small animal model, developing an immuno- *rag^–/–^* myelin-deficient *shi/shi* mouse, in addition to extensive migration of huGRPs, myelination of neonatal mouse brain was also observed^20^. The authors explained differences between the myelinization potential of hGRPs by host species by differing cell transplant microenvironment and immunosuppressive regimens.

Both latter studies suggested, as a next step, the need to develop efficient and safe strategy for cellular graft protection in that specific compartment of the recipient. Moreover, in order to be ready for clinical trials in human subjects, a comprehensive study on the biology of transplanted GRPs, as well as immunoprotective procedures in tested experimental allogenic models, is needed. Pre-clinical small and large animal (mouse and dog, respectively) models should include GRPs both of mouse and dog derivation. In vitro evaluation of the similarities and differences in biological properties between GRPs of mouse, canine, and human species require further careful studies. The expected differences between species, and their CNS immune system interactions, could lead to conclusions regarding possible specific immunomodulatory strategies. Successful strategies for the use of GRPs depend not only on the biological properties of GRPs but also on the immunological microenvironment of damaged CNS, which may influence transplanted cell survival and differentiation potential. Therefore, in this study, we assessed the immunological and functional properties of GRPs of mouse and dog primary cell suspensions, and the human QSV40 cell line, as a prerequisite for a putative experimental pre-clinical model for future ALS treatment.

The cytometric and immunofluorescence analysis of cells in in vitro cultures confirmed the glial-restricted progenitor phenotype of cells in both species examined (mouse and dog) as well as in the human cell line. Purity of GRPs was confirmed by the strong expression of GRP markers such as nestin and A2B5 in the majority of the GRP population. Moreover, the human GRP cell line QSV40 expressed NG2 in addition—a marker specific for glial progenitors. However, GRPs isolated from all species were heterogeneous populations, as confirmed by the presence of small populations of PSA-NCAM^+^ cells specific for mature oligodendrocytes.

One of the biological features of GRPs is an ability to differentiate into astrocytes in culture media enriched in FBS^22,23^. This characteristics was used to confirm GRP biology and the ability to differentiate into mature neural cells. Successful astrocytic differentiation was observed in murine and canine GRPs cell cultures, as proved by positive GFAP expression and negative A2B5 staining. However, no differentiation potential of human GRPs QSV40 immortalized cells into astrocytes was observed. This feature can be explained by the genetic manipulation required to achieve the desired human cell line. Unfortunately, due to local restrictions, in vitro testing of human embryo-derived cells was impossible. However, studies on the differentiation potential of GRPs of human fetal origin into astrocytes have revealed the plasticity of differentiated astrocytes in different culture conditions. The most successful astrocytic differentiation was observed in in vitro cultures supported with FBS or bone morphogenetic protein 4 (BMP-4). In vitro culture supported with ciliary neurotropic factor (CNTF) induced differentiation of human GRPs to an intermediate state, whereas bFGF kept them in an undifferentiated state^22^. These observations may explain the morphological and phenotypic changes in astrocytes in response to inflammatory cytokines upregulated after CNS damage introduced and observed in previous studies^24–26^.

As expected, we confirmed expression of MHC class I and lack of expression of MHC class II antigens on GRPs cells obtained from all the species examined. However, the presence of a low percentage of cells expressing MHC class II (about 1.5%) in murine GRPs and in the human QSV40 cell line can be explained by recent studies suggesting that, in early steps of the developing human brain, MHC-II expression is not restricted to microglia only, but is present in distinct population of neural progenitors and seems to be regulated independently of inflammatory stimuli^27^.

This observation suggests that GRPs had no possibility to participate in immune response as immunocompetent cells. Astrocytic differentiation had no influence on MHC class II expression during in vitro cultures in murine and canine models.

We focused on co-stimulatory molecules CD28, CD40, CD80, and CD154 as molecules associated with antigen-presenting cell function. Our studies documented the lack, or very low number, of cells with expression of CD28, CD80, and CD154 in all the GRPs from different species examined. However, we found that CD40 was present in the majority of GRPs obtained from mouse cells, but the number of GRPs with CD40 expression decreased during long-term in vitro culture. CD40 surface protein is a member of the tumor necrosis factor family^28^, and is present on activated B-cells, making them competent for antigen presentation. This observation suggests that, unlike in large animal models, mouse GRP cells might have the ability to take the function of non-professional antigen-presenting cells. Interestingly, CD40 expression seems to vanish due to the long term in vitro culture of murine GRP cells, which suggests an influence of the in situ environment on the immunological features of GRPs. Expression of CD40 or other co-stimulatory molecules on GRPs was not reported previously. However, in CNS, MHC class II and co-stimulatory molecules (CD40 and CD86 expression) may influence microglia activation, and this process can be regulated differentially depending on the pro-inflammatory millieu^29^.

Neuroinflammation is associated with cytokines and the chemokine activity of microglia and activated glial cells, which release neuroinflammatory mediators leading to neurodegeneration^3,30^. While neuroinflammation associated with microglia activation has been studied and documented widely, there are no reports on the cytokine and trophic factor activity of immature GRPs. Our study documents that substantial levels of MCP-1 are released by canine and murine GRPs. MCP-1 is one of the key chemokines regulating migration and infiltration of monocytes/macrophages, memory T lymphocytes, and natural killer (NK) cells at the injury site. MCP-1 activity plays an important role in normal immune surveillance and immune modulation, and, in the CNS, may protect from acute viral infection. In response to inflammation, MCP-1 attracts monocytes and macrophages to eliminate invading pathogens, as well as in clearing deposits of β-amyloid—a neurotoxic peptide that accumulates in the brain of patients with Alzheimer’s Disease^31^. The low level of MCP-1 released by the human QSV40 cell line is probably associated with manipulation of the cells during the process of immortalization. In contrast, the human QSV40 cell line was effective for GM-CSF production, which functions as a cytokine contributing to the immune/inflammatory cascade. In spinal cord injury models, GM-CSF promotes cortical reactivation and recovery of tactile abilities, but does not influence motor function^32^.

An interesting observation was the production of IL-10 by canine GRPs and the human QSV40 cell line. IL-10 is an immunomodulatory cytokine that may inhibit the activity of proinflammatory cytokines, especially TNFα and IL-6, secreted by activated microglia at the site of neuroinflammation^3^. Secretion of IL-10 by GRPs may enhance the neuroprotective effect of microglia by promotion of the regeneration/immunoregulatory phenotype of M2 macrophages and diminishing the adverse effect of cytotoxic M1 macrophages. Anti-inflammatory chemokines and cytokines MCP-1 and IL-10 are also released at low level (IL-4, IL-13) from GRPs, and may reduce the activity of proinflammatory cytokines and re-establish regenerative processes at the injury site, thus reducing the neurodegeneration process.

We assume that the regenerative potential of GRPs is associated not only with the ability to differentiate into astrocytes but also with the capability to secrete immunomodulatory cytokines, chemokines, and trophic factors (MCP-1, IL-10, GM-CSF), and lack or reduced secretion of proinflammatory cytokines (IFNγ, TNFα, IL-6, IL-12, IL-17). However, canine GRPs and the human cell line QSV40 also produced IL-8—considered a chemotactic and inflammatory cytokine in the neurodegenerative milieu^3^. On the other hand, IL-8 is also known as a potent promoter of angiogenesis when released by mesenchymal stem cells^33^, while, produced by immature GRPs, it might play a role in angiogenic homeostasis.

Stable biological properties of GRPs were maintained during long-term in vitro culture (data not shown). Our observations are in line with studies documenting comparable effects of neurite outgrowth in vitro of all the species analyzed, when GRPs after early and late passage were administered in a rat model of spinal cord injury^34^. However, therapeutic use of GRPs may be limited by the inflammatory microenvironment of the injured CNS, altering the phenotypic and functional properties of any grafted cells. Glial-restricted precursors can be phenotypically sensitive to the inflammatory microenvironment, as confirmed by ex vivo exposure of GRPs to strong inflammatory mediators such as LPS and INFγ. Studies documented that these pro-inflammatory factors altered GRP phenotype and attenuated growth-promoting effects towards neural cells^34^.

## Conclusions

GRPs maintain the stable phenotype of undifferentiated cells in long-term in vitro culture of all the species studied. Canine and human QSV40 cells were immunologically neutral, as proved by lack of expression of MHC-II and co-stimulatory molecules. The presence of CD40 on murine GRPs suggests crucial differences in GRP biology between species. This phenomenon suggests the need for pre-clinical studies with immunosuppressive strategies, including co-stimulatory blockage or calcineurine inhibitors, in different animal models with neurodegenerative diseases. The most clinically relevant animal models of ALS are transgenic mouse overexpressing the mutated SOD1 gene, and a canine model of the disease called degenerative myelopathy, which can deliver significant insights into the mechanisms of motor neuron degeneration in ALS. For studies of Alzheimer’s Disease, transgenic mouse models harboring the human amyloid precursor protein (APP) and Tau mutations, which exhibit intraneuronal and extracellular amyloid pathology and Tau pathology, can be applied.

Phenotypical and immunological characterization of different species GRPs, and the ability for astrocyte differentiation may help to predict GRP responses to the microenvironment of the injured CNS.
